# Implicit and explicit gender identification in autistic and nonautistic gender clinic-referred youth, and their caregivers

**DOI:** 10.1007/s00787-025-02869-5

**Published:** 2025-10-23

**Authors:** Aimilia Kallitsounaki, Matthew C. Fysh, David M. Williams, Lauren Spinner, Eilis Kennedy

**Affiliations:** 1 School of Psychology, University of Kent, Canterbury, UK; 2 Tavistock and Portman NHS Foundation Trust, London, UK; 3 Department of Clinical Educational and Health Psychology, University College London, London, UK; 4 Present Address: Autism Research Centre, Department of Psychiatry, University of Cambridge, Cambridge, UK; 5 Education Endowment Foundation, London, UK

**Keywords:** Autism spectrum disorder, Gender diversity, Implicit association test, Gender identity, Familial aggregation

## Abstract

Gender diversity in autistic youth is sometimes assumed to stem from autism, leading to questions about the authenticity of their gender diverse feelings and experiences. If this were the case, different patterns of gender identity presentation would be expected between autistic and nonautistic gender diverse youth. This hypothesis was examined across two studies. In Study 1, we used an explicit measure together with an Implicit Association Test, for the first time, to examine gender identity in autistic and nonautistic gender-referred and cisgender youth, aged 7–16 years. Gender-referred participants, regardless of autism status, implicitly identified with the binary sex opposite to that assigned at birth and explicitly identified with their experienced gender to a similar degree as their cisgender peers. Autistic cisgender youth also demonstrated implicit and explicit gender identities comparable in strength to their nonautistic peers. In Study 2, we used similar measures to assess gender identification in caregivers. Caregivers of gender-referred youth, regardless of autism status, reported weaker explicit gender-group identification, while implicit identification remained unaffected. Overall, the findings challenge the idea that gender diversity in autism is qualitatively distinct and have important clinical implications for improving support pathways for autistic gender diverse youth.

**Supplementary Information:**

The online version contains supplementary material available at 10.1007/s00787-025-02869-5.

Gender diversity refers to a range of gender identities and expressions that differ from societal and cultural norms typically associated with one’s assigned sex at birth [1]. This includes individuals who identify with a gender opposite to their assigned sex at birth (i.e., transgender) [2], as well as those who experience significant distress about this incongruence (i.e., gender dysphoria) [3]and youth who may be referred to specialist gender identity clinics (henceforth, gender-referred). Twin and family studies have shown that gender diversity may run in families [4–6]. Emerging research suggests that gender diversity frequently intersects with autism spectrum disorder (ASD), a neurodevelopmental condition characterised by difficulties with social-communication and restricted, repetitive patterns of interests and behaviours [3].

Studies of autistic adults and children suggest that the rate of gender diversity in these groups is 7.37% [7], which lies at the very upper end of general population estimates (0.5–8.4%) [8]. It should be noted, however, that research has found significant heterogeneity in the prevalence estimates of gender diversity in autistic samples (range = 0.85%−27.27%) [7]. Cisgender autistic adults also report weaker identification with their assigned sex at birth than nonautistic adults [9, 10], and autism traits in the general population covary with increased feelings of gender dysphoria/diversity and weaker identification with assigned sex at birth [11–15].

It is clear there is a relation between autism and gender diversity, but its nature remains unclear. Some suggest gender diversity in autistic people reflects a specific manifestation of autism, particularly restricted and repetitive behaviours and interests (RRBIs). For instance, gender diversity may represent an obsessive or stereotyped interest in gender, or an overly concrete understanding of it [16]. Alternatively, autistic people may be less influenced by social norms, enabling more open expression of gender diversity than in nonautistic individuals [17]. Despite these hypotheses, little is known about how gender diversity presents in autistic versus nonautistic people. A difficulty with much of the evidence so far is that it is gathered only from self- or parent-report measures of gender identity, which may be biased by social desirability, self-presentation, and/or atypical introspection [18–21]. These issues may be especially relevant in autistic youth, who often show atypical awareness of their own thoughts [22], feelings [23], and bodily states [24].

An alternative approach is to use indirect measures, such as the Implicit Association Test (IAT) [25]. In a gender IAT, participants sort stimuli from four categories (e.g., self, other, female, male) using two response keys. Each response key is associated with a pair of categories (e.g., self and female, or self and male). The task is easier when strongly associated categories share a key than when weakly associated ones do [20]. For example, cisgender individuals typically respond faster when self-related words share a key with sex-congruent terms (i.e., when “self” and “female” share a response key for birth-assigned females and when “self” and “male” share a response key for birth-assigned males), whereas gender diverse individuals respond faster when “self” is paired with sex-incongruent terms [10, 12, 26–32].

Importantly, Kallitsounaki and Williams [10]observed this pattern in both autistic and non-autistic gender diverse adults, suggesting that gender diversity in autism is not merely indexed by self-report but also evident in implicit/indirect measures. Whether this is also true in autistic gender diverse youth remains unknown, as no studies have examined this group. This gap is important, given that gender diversity in autistic youth is sometimes viewed as a byproduct of autism, leading people to question the authenticity of gender diverse experiences in autistic individuals [33]. This perception potentially undermines support [33]and may contribute to a disassociation of autistic gender diverse youth from their autism diagnosis [34], or to increased camouflaging when accessing gender-related services, due to fear of not being recognised as “truly” gender diverse [35].

To address whether gender diversity in autistic youth presents differently in autistic versus nonautistic gender diverse youth we conducted two studies assessing both explicit self-reported and implicit IAT-measured gender identity among autistic and nonautistic, gender-referred and cisgender children and adolescents, as well as their caregivers. Study 1 examined whether gender-referred autistic and nonautistic youth show similar alignment between gender identity and implicit/explicit measures, and whether cisgender autistic youth show weaker gender identity than nonautistic peers. Study 2 investigated whether caregivers of gender-referred youth (autistic and nonautistic) differ from caregivers of cisgender youth in their explicit or implicit gender identity. If gender identity differences show similar patterns within families of both autistic and nonautistic gender-referred youth, that would support the idea that gender diversity in autistic youth is of the same nature or form as in nonautistic youth.

## Study 1: method

### Participants

Two hundred and fifty-nine youth, including autistic gender-referred, nonautistic gender-referred, autistic cisgender, and nonautistic cisgender individuals, were recruited for this study. To be eligible participants had to be aged 7 to 14 years, have a good English language comprehension, be verbally fluent, live in the UK, have parental consent, and have a caregiver who also consented to participate. Additionally, gender-referred youth had to report a gender identity that did not align with their assigned sex at birth, cisgender youth had to report a gender identity that aligned with their assigned sex at birth, and autistic youth had to have a formal diagnosis of autism.

Participants with missing data on the key study variables (*n* = 34), uninterpretable data on the IAT (*n* = 1), or those who were nongender-referred but reported an experienced gender (i.e., the gender that a person identifies with) that did not align with their assigned sex at birth (*n* = 15) were excluded from the analyses.

The final sample included 209 participants aged 7–16 years. To enhance recruitment, participants aged 15 to 16 years were included following an amendment to the study protocol and receipt of the necessary approvals from the relevant ethics boards. Participant characteristics are presented in Table 1. A log-linear analysis indicated no significant differences in assigned sex at birth ratio across groups (*p* =.272, BF_10_= 0.13). However, groups differed significantly in age and FSIQ, as assessed by the Vocabulary and Matrix Reasoning subtests of the WASI-II [36]. To maximise statistical power, we deviated from the pre-registration, and analysed the unmatched samples and reported in this paper. However, matched-sample analyses yielded substantively identical results (i.e., no significant effect observed in the unmatched samples became nonsignificant in the matched samples, or vice versa) and are provided in the Supplementary Information. We would not have deviated from the presentation by using unmatched samples if the results in matched samples differed meaningfully from those in unmatched samples.

**Table 1:** Sample characteristics and two-way ANOVA statistics for age and FSIQ in Study 1

Variable	Autistic		Nonautistic		ANOVA					Direction of Effects
	Gender-referred	Cisgender	Gender-referred	Cisgender						
	*n* = 37 (51% AFAB)	*n* = 55 (42% AFAB)	*n* = 49 (47% AFAB)	*n* = 68 (53% AFAB)						
	*M* (*SD*)	*M* (*SD*)	*M* (*SD*)	*M* (*SD*)	Effect	*F*	*p*	η^2^_p_	BF_10_	
Age	13.57 (2.05)	11.53 (2.10)	11.90 (2.03)	11.18 (2.40)	D	10.71	.001	0.05	4.81	Autistic > Nonautistic
					G	20.00	< .001	0.09	> 30	Gender-referred > Cisgender
					D × G	4.56	.034	0.02	1.33	Cisgender: Autistic = Nonautistic
										Gender-referred: Autistic > Nonautistic
										Autistic: Cisgender < Gender-referred
										Nonautistic: Cisgender = Gender-referred
FSIQ	104.16 (12.17)	103.78 (14.62)	100.92 (13.55)	109.90 (12.08)	D	0.59	.442	< 0.01	0.29	Autistic = Nonautistic
					G	5.32	.022	0.03	3.40	Gender-referred < Cisgender
					D × G	6.30	.013	0.03	3.55	Cisgender: Autistic < Nonautistic
										Gender-referred: Autistic = Nonautistic
										Autistic: Cisgender = Gender-referred
										Nonautistic: Cisgender > Gender-referred
Voc (*t* score)	52.81 (6.59)	52.15 (9.24)	50.94 (10.14)	57.38 (7.62)	D	1.93	.167	0.01	0.74	Autistic = Nonautistic
					G	5.68	.018	0.03	4.22	Gender-referred < Cisgender
					D × G	8.60	.004	0.04	11.42	Cisgender: Autistic < Nonautistic
										Gender-referred: Autistic = Nonautistic
										Autistic: Cisgender = Gender-referred
										Nonautistic: Cisgender > Gender-referred
MR (*t* score)	52.11 (9.15)	52.31 (10.63)	50.27 (8.80)	54.13 (9.24)	D	0.00	.994	< 0.01	0.16	Autistic = Nonautistic
					G	2.28	.133	0.01	0.58	Gender-referred = Cisgender
					D × G	1.85	.175	0.01	0.46	

*Note. N* = 209. *AFAB* = assigned female at birth; *ANOVA* = analysis of variance; *FSIQ* = full scale IQ-2; *Voc* = vocabulary subtest; *MR* = matrix reasoning subtest; *D* = diagnostic status; *G* = gender identity status.

Cisgender participants were recruited through schools, social media, autism charities, and the Kent Child Development Unit database. Gender-referred participants had been referred to a national specialist clinic for gender-related issues and were recruited from an ongoing prospective longitudinal study [37] and social media. Out of 86 gender-referred participants, 80 reported a gender identity that did not align with their assigned sex at birth (76 identified with the binary gender opposite to their assigned sex at birth, and 4 identified as both genders). The remaining six participants reported a gender identity that was congruent with their assigned sex at birth. Excluding these six participants results did not change substantively (see Supplementary Information), so they were included in the main analyses, deviating from the pre-registration to maximise statistical power. It is also important to note that these six gender-congruent participants were referred to a specialist clinic, reflecting the presence of significant concerns about gender identity even if the participant reported a currently congruent identity. Such fluidity over time may be a hallmark of gender diversity/dysphoria, so excluding those participants risks producing unrepresentative groups (though we stress we would have removed them if results changed meaningfully after their exclusion, which they did not). Nearly all participants (99.04%) were native English speakers.

All participants in the autism groups had a formal diagnosis of ASD (*M*_age of diagnosis_ = 8.76, *SD* = 2.83; *n* = 85), with the exception of five individuals who were in the process of assessment. Excluding these participants did not change the results (see Supplementary Information), so they were retained in the main analyses, deviating from the pre-registration to maximise statistical power. Autism features were assessed in all but eight autistic participants via the Brief Observation of Symptoms of Autism (BOSA) [38, 39]and in all but two via the Autism Diagnostic Interview-Revised (ADI-R) [40]. The BOSA is an observational diagnostic measure, derived from the Autism Diagnostic Observation Schedule–2 (ADOS-2) [41], designed for online/virtual administration. The ADI-R is a standardised, comprehensive diagnostic interview conducted with caregivers to assess their child’s early development and current behaviour. 96% of participants scored above the clinical cut-off on either the BOSA (≥ 6) or on all three ADI-R Domains: A (≥ 10), B (≥ 8), and C (≥ 3). Breaking this down, 85% of participants scored above the clinical cut-off on the BOSA (≥ 6) and a high percentage of participants also exceeded the clinical cut-offs on each domain of the ADI-R: 90% scored ≥ 10 in Domain A (Social Interaction), 91% scored ≥ 8 in Domain B (Communication), and 93% scored ≥ 3 in Domain C (Restricted and Repetitive Behaviours).

Prior to participation in the study, written electronic informed consent was obtained from all caregivers for their child’s participation, and written electronic informed assent was obtained from all participating children and young people. The study was approved by the Kent Psychology (approval number: 202216553002907588) and HRA and London–Hampstead (reference number: 22/LO/0805) Research Ethics Committees. The study was preregistered on Open Science Framework (preregistration can be viewed here: https://osf.io/u3xvm/?view_only=70e0a169251e49b9bf5aa6b6ffa26e2c). Deviations from the preregistration are presented in the Supplementary Information.

### Materials

#### Gender identity IAT

To measure implicit gender self-concept, we used the Gender Identity IAT employed by Gülgöz et al. [29]; following Olson et al. [30]. Children classified words and pictures using two response keys (A and L) into one of four categories: (a) Me [I, me, mine, myself]; (b) Not Me [they, them, theirs, other]; (c) Girl [four pictures of girls]; and (d) Boy [four pictures of boys]. The pictures of girls and boys were selected from the National Institute of Mental Health Child Emotional Faces Picture Set (NIMH-ChEFS) [42] and depicted neutral child faces with a direct gaze.

**Fig. 1:**
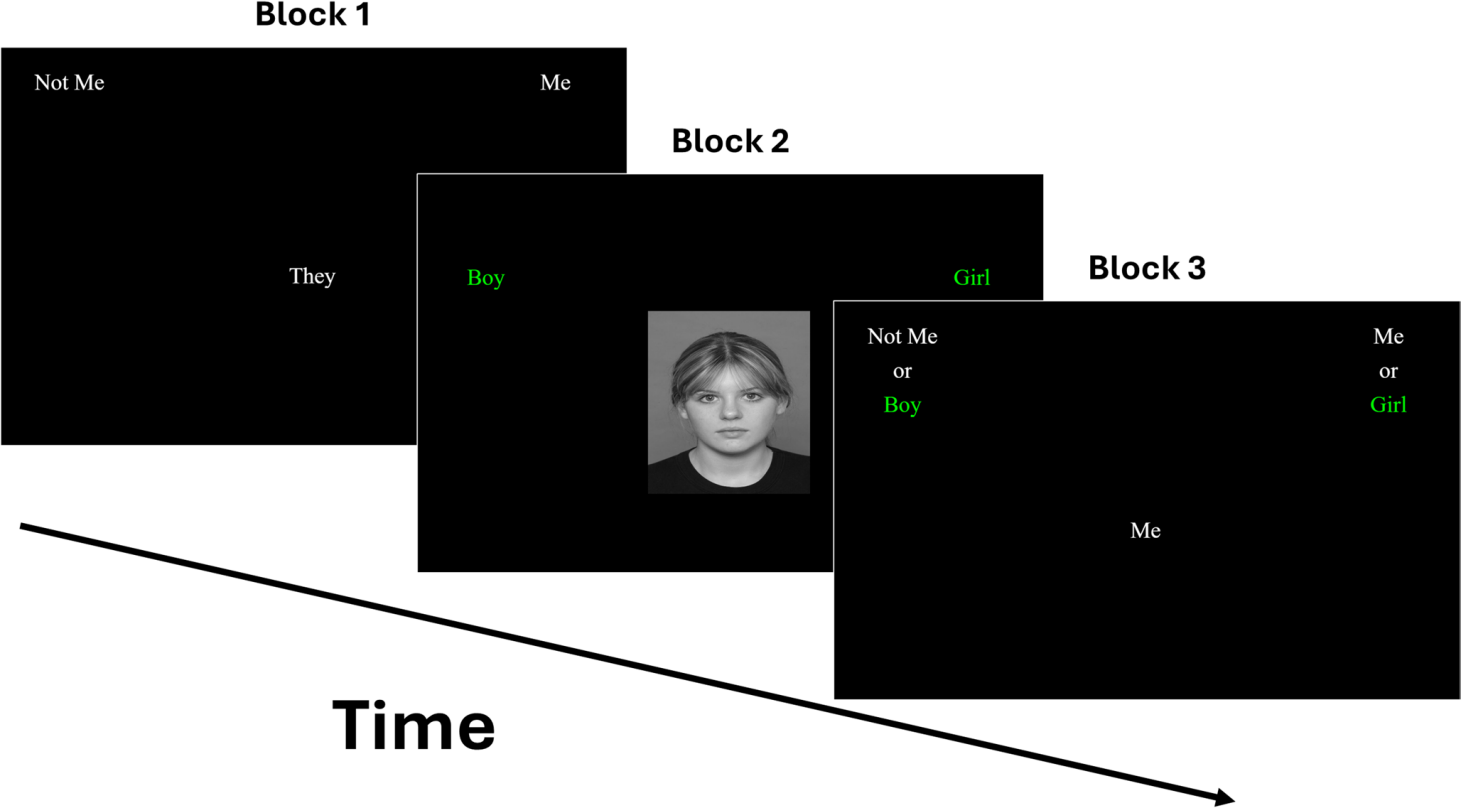
Illustration of Blocks 1 (Me vs. Not Me), 2 (Boy vs. Girl) and 3 (Me/Girl vs. Not Me/Boy) of the IAT in Study 1. Note. In this example, block 5 would be identical to block 1, except with the *Me* and *Not Me* categories swapping sides. Blocks 6 and 7 would be identical to block 3, but with the *Me* and the *Not Me* categories again on opposite sides

The task comprised seven blocks [20], of which an example of the first three blocks is shown in Fig.1. Blocks 1 and 2 introduced the binary categories separately (Me vs. Not Me; Boy vs. Girl) across 20 trials each. Blocks 3 and 4 presented combined categories (e.g., Me/Girl; Not Me/Boy), with block 3 as practice and block 4 comprising 40 experimental trials. In Block 5, the Me/Not Me categories switched sides. Blocks 6 and 7 repeated the combined sorting with the reversed Me/Not Me positions (e.g., Me/Boy; Not Me/Girl). The sequence of combinations in blocks 3–7 was counterbalanced across participants. To proceed from one trial to the next, participants had to provide a correct categorisation. In cases of incorrect responses, a red “X” appeared on the screen, and participants were instructed to correct their mistake in order to continue.

Automatic association strength (D score) was calculated using Greenwald et al.'s algorithm [43]. This algorithm uses data from the critical blocks (blocks 3, 4, 6, and 7) only, and requires trials with latencies > 10,000 ms as well as participants with > 10% of trials < 300ms to be excluded (*n* = 1). Two standard deviations were calculated: one from the practice blocks (3 and 5), and another from the experimental blocks (4 and 7). Mean response times were then computed for each of these four blocks (3, 4, 6, and 7). Two difference scores were then calculated by subtracting the mean latency of block 6 from block 3, and the mean latency of block 7 from block 4. Each difference score was then divided by its corresponding standard deviation. Lastly, the two resulting values were averaged to yield the overall IAT D score.

Participants who responded faster in the Me/Girl condition than in the Me/Boy condition received a positive D score, indicating a female self-concept. Conversely, participants who responded faster in the Me/Boy condition than in the Me/Girl condition received a negative D score, indicating a male self-concept (i.e., intergroup scoring).

For the main analysis, we used an alternative method in which positive scores reflect a stronger association with one’s assigned sex at birth, and negative scores reflect a stronger association with the opposite sex (e.g., ingroup scoring). To achieve this, D scores remained the same for participants assigned female at birth, whereas positive and negative scores were reversed for participants assigned male at birth (i.e., positive scores were converted to negative scores and negative scores were converted to positive scores).

Task accuracy was calculated as the proportion of correct responses in the critical blocks (i.e., blocks 3–4, and 6–7). The task was programmed in PsychoPy (v2021.1.4) [44] and administered online via Pavlovia.

#### Explicit gender identity

To measure explicit gender self-concept, participants who reported a binary gender identity were asked, “How much like a boy/girl do you feel?” using a 3-point scale (“a little bit,” “moderately,” “very much”). The gender label matched the participant’s gender identity. Scores ranged from 1 to 3, with higher scores indicating a stronger explicit gender self-concept.

Participants who identified as nonbinary were asked two separate questions: one about feeling like a girl and another about feeling like a boy. Scores from these two questions were averaged to create a composite explicit gender self-concept score. Gender-referred participants who identified as genderfluid, questioning, or agender (*n*= 4) did not complete this task. Note that this single-item measure was developed by the authors based on current conceptualisations of gender in research [45]and on how gender is often described by gender diverse people, emphasising the internal experience of gender rather than characteristics associated with assigned sex at birth [46].

### Procedure

The majority of participants completed the Matrix Reasoning and Vocabulary subtests of the WASI-II, along with the IAT and the explicit task, during a virtual session with a researcher. A small subset of participants (7 out of 208) completed these tasks either in person or independently via Qualtrics. Following Nosek at al.’s [20] recommendation, all participants completed the IAT and the explicit task in counterbalanced order.

### Statistical analysis

To examine between-group differences in IAT performance, we performed a series of analyses, each with a different dependent variable. First, we conducted a 2 (diagnostic status: autistic\non-autistic) × 2 (gender identity status: gender-referred/cisgender) ANOVA on the proportion of correct responses in the critical blocks (3, 4, 6, and 7) to assess group differences in IAT accuracy. Second, we conducted a 2 × 2 ANOVA on IAT D scores to assess group differences in the strength of the IAT automatic association. Third, we conducted a series of one-sample *t*-tests on IAT D scores to examine whether the strength of the automatic association was significantly above chance level in each gender identity group. Fourth, we conducted a 2 × 2 ANOVA on scores from the explicit gender identity task to assess group differences in the strength of explicit gender identity.

Effect sizes are reported using (a) partial eta squared (η_p_²) with 0.01, 0.06, and 0.14 indicating small, medium, and large effects, respectively, or (b) Cohen’s *d*, with 0.20, 0.50, and 0.80 indicating small, medium, and large effects, respectively. Bayesian analyses assessed evidence strength for the alternative versus null hypothesis [47]. Bayes factors (BF₁₀) > 1 support the alternative hypothesis (with > 3, >10, and > 30 reflecting substantial to very strong evidence), while values < 1 support the null (< 0.33, < 0.10, and < 0.03 indicating substantial to very strong evidence). Analyses were conducted in R (v4.3.3) and JASP (v0.19.3).

## Study 1: results

### Implicit gender

A preliminary 2 (diagnostic status: autistic/nonautistic) × 2 (gender identity status: gender-referred/cisgender) ANOVA was conducted on the proportion of correct responses in the critical IAT blocks (see Table 2). A significant main effect of gender identity status indicated that gender-referred participants (*M* = .95; *SD* = .04) performed significantly better than cisgender participants (*M* = .93; *SD* = .06). Neither the main effect of diagnostic status nor the Diagnostic Status × Gender Identity Status interaction was significant.

**Table 2:** Mean scores and two-way ANOVA statistics for IAT and explicit task performance in Study 1

Variable	Autistic		Nonautistic		ANOVA					Direction of Effects
	Gender-referred	Cisgender	Gender-referred	Cisgender						
	*M *(*SD*)	*M *(*SD*)	*M *(*SD*)	*M *(*SD*)	Effect	*F*	*p*	η^2^_p_	BF_10_	
IAT correct	.94 (.04)	.92 (.05)	.95 (.03)	.93 (.06)	D	2.80	.096	.01	0.56	Autistic = Nonautistic
					G	7.45	.007	.04	5.96	Gender-referred >Cisgender
					D × G	0.10	.757	< .01	0.22	
IAT D	−0.14 (0.34)	0.34 (0.29)	−0.20 (0.31)	0.29 (0.31)	D	1.48	.225	.01	0.27	Autistic = Nonautistic
					G	118.28	< .001	.37	>30	Gender-referred < Cisgender
					D × G	0.01	.944	< .01	0.21	
Explicit	2.74 (0.52)	2.84 (0.37)	2.84 (0.37)	2.81 (0.43)	D	0.30	.582	< .01	0.16	Autistic = Nonautistic
					G	0.30	.586	< .01	0.17	Gender-referred = Cisgender
					D × G	1.02	.313	.01	0.31	

*Note.* *N* = 209. ANOVA = analysis of variance; D = child diagnostic status; G = child gender identity status; IAT correct = Implicit Association Test proportion of correct responses in the critical blocks; IAT D = Implicit Association Test D score.

A second 2 (diagnostic status) × 2 (gender identity status) ANOVA was conducted on D scores (see Table 2). A significant main effect of gender identity status was again observed, with gender-referred participants scoring significantly lower (*M* = −0.17; *SD* = 0.32) than cisgender participants (*M* = 0.31; *SD* = 0.30). This suggests that gender-referred participants identified less strongly with their assigned sex at birth. Neither the main effect of diagnostic status nor the interaction was significant.

One-sample *t*-tests showed that gender-referred participants scored significantly below zero, *t*(85) = −4.90, *p* < .001, *d* = −0.53, BF_10_ > 30, indicating a stronger identification with the opposite than same binary sex. In contrast, cisgender participants scored significantly above zero, *t*(122) = 11.38, *p* < .001, *d* = 1.03, BF_10_ > 30, indicating a stronger identification with their birth-assigned than opposite sex.^1^

### Explicit gender

A 2 (diagnostic status: autistic/nonautistic) × 2 (gender identity status: gender-referred/cisgender) ANOVA was conducted on participants’ scores from the explicit measure (see Table 2). No effects were significant, indicating that participants reported similar levels of identification with the experienced gender, regardless of their gender identity or diagnostic status.

## Study 1: discussion

No significant between-group differences were found in the extent to which participants explicitly identified with their experienced gender. Gender diverse participants identified with their experienced gender to the same degree that cisgender participants identified with their assigned sex at birth, regardless of autism status. Similarly, IAT performance aligned with experienced gender across all participant groups. Critically, the magnitude and direction of IAT D scores did not differ between autistic and nonautistic gender diverse participants. Thus, our findings provide no support for the idea that gender identity operates differently in autistic versus nonautistic gender diverse youth.

In Study 2, we examined explicit and implicit gender identity among caregivers of the participants from Study 1. The primary aim was to assess whether gender identity strength differed between caregivers of autistic and nonautistic gender diverse youth. Autism is known to run in families [48], and even nonautistic relatives often exhibit elevated autism traits, a phenomenon known as the broad autism phenotype [49, 50]. However, whether a similar “broad gender phenotype” exists, and whether it differs between families of autistic and nonautistic individuals, remains unclear. If reduced identification with assigned sex at birth were observed only in caregivers of nonautistic gender diverse youth, this would support the hypothesis that gender identity variation is distinct in autism.

## Study 2: method

### Participants

Two hundred and fifty-nine caregivers of autistic gender-referred, nonautistic gender-referred, autistic cisgender, and nonautistic cisgender youth participated in this study. Participants were excluded from the analyses if they had missing data on key study variables (*n* = 27), or if their child reported a noncisgender identity despite not being from the gender-referred group (*n* = 14), or if their child did not provide information about their gender identity (*n* = 1). Final sample characteristics are in Table 3. Fisher’s exact tests revealed a significantly higher proportion of assigned male at birth caregivers in the nonautistic cisgender group compared to the autistic gender-referred group (*p* = .006, BF_10_ = 13.44). No other between-group differences in caregiver sex ratios were statistically significant (all *p*s ≥ .195, BF_10_s ≤ 1.18). Groups also differed in age (see Table 3). However, when groups were matched for age and assigned sex at birth, results from the main analysis did not change substantively (see Supplementary Information). 96% of participants were birth parents,^2^ 99.07% reported being cisgender, and 91.24% reported English as their first language. Recruitment and ethics procedures were consistent with those described in Study 1.

**Table 3:** Sample characteristics and two-way ANOVA statistics for age, as well as IAT and explicit task performance in study 2

Variable	Autistic		Nonautistic		ANOVA					Direction of Effects
	Gender-referred	Cisgender	Gender-referred	Cisgender						
	*n* = 49 (98% AFAB)	*n* = 54 (93% AFAB)	*n* = 51 (90% AFAB)	*n* = 63 (81% AFAB)						
	*M* (*SD*)	*M* (*SD*)	*M* (*SD*)	*M* (*SD*)	Effect	*F*	*p*	η^2^_p_	BF_10_	
Age	44.92 (4.62)	42.70 (6.41)	44.69 (6.78)	43.19 (4.36)	D	0.03	.868	< 0.01	0.15	Autistic = Nonautistic
					G	5.90	.016	0.03	2.25	Gender-referred > Cisgender
					D × G	0.22	.638	< 0.01	0.76	
IAT correct	.97 (.15)	.99 (.13)	.97 (.02)	.97 (.03)	D	0.51	.477	< 0.01	0.19	Autistic = Nonautistic
					G	0.96	.328	< 0.01	0.22	Gender-referred = Cisgender
					D × G	0.32	.570	< 0.01	0.23	
IAT D	0.45 (0.44)	0.52 (0.38)	0.52 (0.29)	0.57 (0.35)	D	1.57	.211	0.01	0.32	Autistic = Nonautistic
					G	1.54	.216	0.01	0.32	Gender-referred = Cisgender
					D × G	0.05	.817	< 0.01	0.21	
Explicit	4.04 (1.98)	4.73 (1.23)	4.53 (1.13)	4.78 (1.01)	D	2.00	.158	0.01	0.36	Autistic = Nonautistic
					G	6.44	.012	0.03	2.84	Gender-referred < Cisgender
					D × G	1.43	.233	0.01	0.37	

*Note. N* = 217. *AFAB* = assigned female at birth; *ANOVA* = analysis of variance; *D* = child diagnostic status; *G* = child gender identity status; *IAT correct* = Implicit Association Test proportion of correct responses in the critical blocks; *IAT D* = Implicit Association Test D score.

### Measures

#### Gender identity IAT

To assess implicit gender self-concept, we used the Implicit Association Test (IAT) described by Greenwald et al. [28]. The task design mirrored Study 1, except participants sorted only words into categories. The categories and stimuli were: (a) Self [I, me, my, mine, self]; (b) Other [they, them, their, it, other]; (c) Female [woman, girl, daughter, madam, lady, female]; and (d) Male [man, boy, son, sir, gentleman, male]. All other aspects of the task, including counterbalancing, block order, and scoring procedures, were identical to Study 1.

#### Explicit gender identity

To assess explicit gender-group identification, participants rated each of the six male and six female nouns from the IAT on a 7-point scale, ranging from “not at all characteristic of me” to “extremely characteristic of me” [28]. Scores were calculated by subtracting the average rating for male nouns from the average rating for female nouns. For preliminary analyses, intergroup scoring was applied, with positive scores indicating stronger identification with the female group and negative scores with the male group. This scoring was necessary to confirm that the explicit gender identity task was sensitive to assigned sex at birth differences (i.e., participants assigned female at birth scoring higher than participants assigned male at birth) and to validate that it measured the intended construct. For the main analyses, ingroup scoring was used, where positive scores reflected identification with the gender group aligned with the participant’s assigned sex at birth and negative scores reflected identification with the opposite binary sex. This scoring was necessary to examine between-group differences in the strength of explicit gender identity.

### Procedure

Participants completed all tasks independently online via Qualtrics for the explicit task and Pavlovia for the IAT. Following Nosek at al.’s [20] recommendation, all participants completed the IAT and the explicit task in counterbalanced order.

### Statistical analysis

To examine between-group differences in IAT performance, we performed a series of analyses, each with a different dependent variable. First, we conducted a series of *t*-tests to assess whether the IAT was sensitive to assigned sex at birth differences. For these preliminary analyses, intergroup scores were used. Second, we conducted a 2 (child diagnostic status: autistic/nonautistic) × 2 (child gender identity status: gender-referred/cisgender) ANOVA on the proportion of correct responses in the critical blocks (3, 4, 6, and 7) to assess group differences in IAT accuracy. Third, we conducted a 2 × 2 ANOVA on IAT D scores to assess group differences in the strength of the IAT automatic association. Note for this analysis ingroup scores were used.

To examine between-group differences in the explicit gender identity task we performed a series of analyses, each with a different dependent variable. First, we conducted a series of *t*-tests to assess whether the explicit gender identity task was sensitive to assigned sex at birth differences. For these preliminary analyses, intergroup scores were used. Second, we conducted a 2 × 2 ANOVA on scores from the explicit gender identity task to assess group differences in the strength of explicit gender identity. For this analysis, ingroup scores were used.

## Study 2: results

### Implicit gender

One-sample tests showed that D scores differed significantly from zero (i.e., a neutral gender self-concept) in participants assigned female at birth (*n* = 195; *M* = 0.52; *SD* = 0.37), *t*(194) = 19.92, *p* < .001, *d* = 1.43, BF_10_ > 30, as well as participants assigned male at birth (*n* = 22; *M* = −0.49; *SD* = 0.38), *t*(21) = −6.00, *p* < .001, *d* = −1.28, BF_10_ > 30. Moreover, an independent *t*-test showed that the difference in D score between sexes was significant, *t*(215) = 12.17, *p* < .001, *d* = 2.74, BF_10_ > 30, confirming that participants assigned female at birth demonstrated a stronger association with female gender, while participants assigned male at birth showed a stronger association with male gender.

A 2 (child diagnostic status: autistic/nonautistic) × 2 (child gender identity status: gender-referred/cisgender) ANOVA was then conducted on IAT D scores (see Table 3, which also shows groups were equivalent in percentage of correct responses on critical blocks). Neither main effect nor the Child Diagnostic Status × Child Gender Identity Status interaction was significant, suggesting that caregivers’ implicit identification with their assigned sex at birth did not differ significantly by children’s gender identity or diagnostic status.

### Explicit gender

First, one-sample *t*-tests showed that self-reported strength of gender identity differed significantly from zero in participants assigned female at birth (*n* = 195; *M* = 4.51; *SD* = 1.42), *t*(194) = 44.31, *p* < .001, *d* = 3.17, BF_10_ > 30, as well as participants assigned male at birth (*n* = 22; *M* = −4.77; *SD* = 0.95), *t*(21) = −23.62, *p* < .001, *d* = −5.04, BF_10_ > 30. An independent *t*-test showed that the difference between sexes was significant, *t*(215) = 29.85, *p* < .001, *d* = 6.71, BF_10_ > 30.

A 2 (child diagnostic status: autistic/nonautistic) × 2 (child gender identity status: gender-referred/cisgender) ANOVA was then conducted on participants’ explicit task scores (see Table 3). This revealed a significant main effect of child gender identity status, with caregivers of gender-referred youth (*M* = 4.51; *SD* = 1.52) reporting significantly lower gender-group identification than caregivers of cisgender youth (*M* = 4.84; *SD* = 1.05). Neither the main effect of child diagnostic status nor the interaction was significant.

## Study 2: discussion

While all caregiver groups identified relatively strongly with their experienced gender (which, for most, aligned with assigned sex at birth), explicit gender identity was significantly weaker in caregivers of gender diverse youth compared to those of cisgender youth. Importantly, this effect was unaffected by the child’s diagnostic status. Caregivers of autistic and nonautistic cisgender youth reported equivalent gender identity strength, as did caregivers of autistic and nonautistic gender diverse youth.

In contrast, child gender identity had no effect on caregivers’ implicit gender identity. IAT performance aligned with assigned sex at birth across all caregiver groups, with no between-group differences in the strength of this alignment. Thus, while caregivers of gender-diverse youth reported weaker explicit gender identity, this was not reflected in their IAT performance.

## General discussion

Across two studies, we found consistent evidence that gender identity presentation is equivalent in autistic and nonautistic youth, and their families. In Study 1, we replicated Gülgz et al. [29] finding that implicit gender identity, measured via a gender IAT, aligns with experienced gender identity in nonautistic gender diverse youth. Crucially, we observed the same alignment in autistic gender diverse youth. This provides the first evidence that autistic and nonautistic gender diverse youth alike exhibit both explicit and implicit gender self-concepts that reflect their experienced gender identity. These findings strengthen the argument that gender identity variation occurs similarly across autistic and nonautistic gender diverse populations.

We also found that cisgender autistic youth were equivalent to their nonautistic peers in both self-reported gender identity strength and IAT performance. This contrasts with prior research in cisgender adults, where autistic individuals have shown weaker explicit gender identity than nonautistic adults [10]. One possibility is that such differences emerge over time, with gender identity consolidating less in autistic than in nonautistic individuals. Longitudinal studies are needed to test this hypothesis. For now, our results indicate that cisgender autistic youth do not experience a diminished sense of gender identity, so clinicians should not assume atypical gender identity based on autism diagnosis alone.

Study 2 offered novel evidence that subtle gender identity differences are also present in caregivers of gender diverse youth. Caregivers of both nonautistic and autistic gender diverse children reported significantly weaker explicit identification with their assigned sex at birth than caregivers of cisgender youth. This suggests that gender identity variation may aggregate in families, although whether this reflects genetic or environmental mechanisms remains unclear. Importantly, child diagnostic status had no effect on this pattern, indicating similar familial aggregation in both autistic and nonautistic groups. This finding challenges the idea that gender diversity in autism is qualitatively distinct. If it were, differing patterns of familial aggregation might be expected.

Interestingly, while explicit gender identity aggregated within families, implicit gender identity did not. The reason for this discrepancy is unclear. One possibility is that the IAT is sensitive to significant identity variation but less capable of detecting subtle shifts captured by self-report. Given the IAT’s lower test-retest reliability than self-report [51], measurement noise may also play a role. Alternatively, caregivers of gender diverse children may distance themselves from traditional gender roles because of their parenting experiences, which might shape self-reports more than implicit association patterns. Regardless, it is important to note that that almost all of the caregivers in Study 2 were assigned female at birth, so it remains uncertain whether similar familial aggregation would appear in birth-assigned male caregivers. Nonetheless, our findings show that explicit gender identity variation is similarly expressed among female caregivers of both autistic and nonautistic gender diverse youth.

### Limitations and directions for future research

As already noted, this study includes several deviations from the pre-registration (see Supplementary Information for details), including the inclusion of participants under autism assessment in the autistic group, participants who had been referred to a gender identity clinic for gender issues yet still reported a gender identity congruent to their assigned sex at birth in the gender-referred group, and lack of matching across groups for age and intellectual abilities. These deviations could potentially bias the results in a number of different ways. For example, including participants under assessment may deflate autistic group scores in the gender identity tasks leading to nonsignificant between-group differences. To address these deviations, all analyses were repeated following our pre-registered plan, and no meaningful differences in the results were found. Hence, when we conducted analyses entirely in line with pre-registration, the results were equivalent to when analyses deviated from the plan, reassuring us that the deviations did not significantly impact our findings.

While the final sample (*N* = 209) slightly exceeded the pre-registered target (*N* = 200), recruitment challenges prevented us from reaching the planned sample size in the gender-referred groups. To maximise power, we raised the upper age limit in Study 1 from 14 to 16 years, yielding nearly the intended sample in the gender-referred groups (*n* = 49 nonautistic and 37 autistic unmatched; 35 per group matched). Although still below the pre-registered level of 50 per group, the absence of diagnostic group differences was unlikely due to low power. Diagnostic group differences (including interaction effects) were associated with small-to-minuscule effect sizes and Bayes factors that supported the null hypothesis in every case, suggesting the lack of group differences does not reflect Type II error.

A standard note of caution should also be added regarding the generalisability of the findings from Studies 1 and 2. It is unclear, whether the same results would be observed in youth with autism who have cooccurring intellectual difficulties or gender diverse youth from a nonclinic population. Additionally, the fact that participants were from the UK and most were native English language speakers limits the extent to which firm conclusions can be drawn about the presentation of gender diversity in autistic youth and their caregivers from different cultural backgrounds.

It is important to note that the use of IATs to measure individual differences has been questioned, given their modest test–retest reliability (approximately 0.50 across domains [52]; 0.34–0.48 for gender identity IATs [53]). Although this level of stability precludes their use as diagnostic tools, it is generally considered sufficient for testing hypotheses about group differences, as in the current study [52]. Consistent with this, the present findings and prior research show that the gender identity IAT reliably distinguishes cisgender from gender diverse participants, supporting its validity in capturing individual differences in gender identity. Nonetheless, future work should examine whether the gender IAT functions similarly for nonbinary individuals, given its binary structure and the inclusion of pronouns in the “Other” category (e.g., they, them) that nonbinary participants may use for self-reference. Future research could also incorporate additional indirect measures of gender identity, such as the self-reference task employed by Gülgöz et al. [54], to provide complementary evidence and further assess the validity of the IAT.

Lastly, although this study shows that implicit and explicit gender identification are similarly strong in autistic and nonautistic youth, different factors may contribute to gender diversity in the two groups. Future research could explore this possibility by examining potential contributing factors, such as differing developmental trajectories in gender identity, consistent with the principle of equifinality, where similar outcomes may arise from different pathways.

In general, the current results are consistent with recent findings of broad similarities in gender phenotype between gender diverse youth with and without autism [55]and with high versus low autistic traits [56]. While not definitive, these findings reinforce the validity of experienced gender identity in autistic youth and challenge assumptions that such identities result from autism itself. This is particularly important in the context of clinical assessments, where autistic youth could face additional scrutiny in accessing support for their gender identity issues care.

## Supplementary Information

Below is the link to the electronic supplementary material.

Supplementary Material 1 (DOCX 68.7 KB)

## Data Availability

Data will be made available on Open Science Framework upon publication.

## Footnotes

1 Note that matching the groups on the proportion of correct responses in the critical blocks did not alter the results of the D-score analyses (see Supplementary Information).

2 The main aim of Study 2 was to explore whether gender identity variation aggregates differently in families of autistic versus nonautistic gender-referred youth. Determining the extent to which any such aggregation is driven by genetic versus environmental factors would require twin or adoption studies. As such, caregivers participated in this study regardless of their relationship to the young person.
