# Supplementary material for: Implicit and explicit gender identification in autistic and nonautistic gender clinic-referred youth, and their caregivers
Source: Eur Child Adolesc Psychiatry. 2025 Oct 23;35(3):857–68. doi: 10.1007/s00787-025-02869-5 (PMC13212394; doi:10.1007/s00787-025-02869-5)
Supplement: Supplementary file 1 — Supplementary Material 1 (DOCX 68.7 KB) [file 787_2025_2869_MOESM1_ESM.docx]

**Supplementary Information**

**Study 1: Results After Matching Groups for Age and FSIQ**

To ensure that the findings of Study 1 could not be explained by between-group differences in age and FSIQ, we matched groups on these variables using an R script (version 4.3.3; R Core Team, 2024). Groups were considered matched if *p*-values were greater than .05 and effect sizes were small. To achieve this, we followed a series of steps.

In Step 1, the youngest participants from the autistic cisgender, nonautistic cisgender, and nonautistic gender-referred groups were gradually removed until the sex ratio and group sizes (*n*s) were balanced with those of the autistic gender-referred group. When participants within the same group had the same age, ties were resolved by excluding the participant with the lowest FSIQ in the autistic cisgender and nonautistic gender-referred groups, and the participant with the highest FSIQ in the nonautistic cisgender group. After this step, analyses showed that the groups were matched for FSIQ but remained unmatched for age, so we proceeded to the next step.

In Step 2, the oldest participant from the autistic gender-referred group and the youngest participants from the other groups, regardless of FSIQ or assigned sex at birth, were excluded. After this step, analyses showed that groups were still unmatched for age, so we proceeded to the final step.

In Step 3, we repeated the procedure followed in Step 2. Analyses showed that groups were now matched for FSIQ, age, and assigned sex at birth. The final matched sample characteristics and matching statistics are reported in Table S1.

| **Table S1** | | | | | | | | | | |
| --- | --- | --- | --- | --- | --- | --- | --- | --- | --- | --- |
| *Sample Characteristics and Matching Statistics for Age and FSIQ in Study 1* | | | | | | | | | | |
| Variable | Autistic | | Nonautistic | | ANOVA | | | | | Direction of Effects |
|  | Gender-referred | Cisgender | Gender-referred | Cisgender |  |  |  |  |  |  |
|  | *n* = 35  (54% AFAB)^a^ | *n* = 35  (49% AFAB) | *n* = 35  (49% AFAB) | *n* = 35  (49 % AFAB) |  |  |  |  |  |  |
|  | *M* (*SD*) | *M* (*SD*) | *M* (*SD*) | *M* (*SD*) | Effect | *F* | *p* | η^2^_p_ | BF_10_ |  |
| Age | 13.43 (2.02) | 12.71 (1.54) | 12.89 (1.37) | 13.11 (1.23) | D | 0.07 | .788 | < .01 | 0.19 | Autistic = Nonautistic |
|  |  |  |  |  | G | 0.84 | .361 | .01 | 0.27 | Gender-referred = Cisgender |
|  |  |  |  |  | D × G | 3.16 | .078 | .02 | 0.94 |  |
| FSIQ | 103.74 (12.02) | 104.54 (15.45) | 101.17 (13.83) | 104.74 (10.17) | D | 0.29 | .591 | < .01 | 0.21 | Autistic = Nonautistic |
|  |  |  |  |  | G | 0.99 | .322 | .01 | 0.29 | Gender-referred = Cisgender |
|  |  |  |  |  | D × G | 0.40 | .530 | < .01 | 0.28 |  |
| Voc (*t* score) | 52.80 (6.56) | 52.20 (9.92) | 51.06 (10.20) | 53.97 (6.33) | D | 0.00 | .992 | < .01 | 0.18 | Autistic = Nonautistic |
|  |  |  |  |  | G | 0.66 | .419 | .01 | 0.25 | Gender-referred = Cisgender |
|  |  |  |  |  | D × G | 1.51 | .221 | .01 | 0.47 |  |
| MR (*t* score) | 51.63 (9.00) | 53.06 (10.87) | 50.40 (9.06) | 51.63 (7.89) | D | 0.72 | .398 | .01 | 0.25 | Autistic = Nonautistic |
|  |  |  |  |  | G | 0.72 | .398 | .01 | 0.25 | Gender-referred = Cisgender |
|  |  |  |  |  | D × G | 0.00 | .949 | < .01 | 0.23 |  |
| *Note.* *N* = 140. AFAB = assigned female at birth; ANOVA = analysis of variance; FSIQ = full scale IQ-2; Voc = vocabulary subtest; MR = matrix reasoning subtest; D = diagnostic status; G = gender identity status.  ^a^ Groups were matched for assigned sex at birth (*p* = .735, BF_10_ = 0.14). | | | | | | | | | | |

Table S2 shows the mean scores on the IAT and the explicit task, along with the corresponding ANOVA results. As in the unmatched sample, a 2 (diagnostic status: autistic/nonautistic) × 2 (gender identity status: gender-referred/cisgender) ANOVA on the proportion of correct responses in the critical IAT blocks yielded a significant main effect of gender identity status, indicating that gender-referred participants (*M* = .95; *SD* = .04) performed significantly better than cisgender participants (*M* = .93; *SD* = .06). Neither the main effect of diagnostic status nor the Diagnostic Status × Gender Identity Status interaction was significant.

Consistent with the unmatched sample, a 2 (diagnostic status: autistic/nonautistic) × 2 (gender identity status: gender-referred/cisgender) ANOVA on D scores yielded a significant main effect of gender identity status, with gender-referred participants (*M* = -0.15; *SD* = 0.33) scoring significantly lower than cisgender participants (*M* = 0.31; *SD* = 0.28). Neither the main effect of diagnostic status nor the interaction was significant. Furthermore, one-sample *t*-tests showed that, as in the unmatched sample, gender-referred participants scored significantly below zero, *t*(69) = -3.81, *p* < .001, *d* = -0.46, BF_10_ > 30, whereas cisgender participants scored significantly above zero, *t*(69) = 9.11, *p* < .001, *d* = 1.09, BF_10_ >30.

Lastly, as in the unmatched sample a 2 (diagnostic status: autistic/nonautistic) × 2 (gender identity status: gender-referred/cisgender) ANOVA on scores from the explicit measure yielded no significant main effects or interaction.

| **Table S2** | | | | | | | | | | |
| --- | --- | --- | --- | --- | --- | --- | --- | --- | --- | --- |
| *Mean Scores and ANOVA Results After Matching Groups* | | | | | | | | | | |
| Variable | Autistic | | Nonautistic | | ANOVA | | | |  | Direction of Effects |
|  | Gender-referred | Cisgender | Gender-referred | Cisgender |  |  |  |  |  |  |
|  | *M* (*SD*) | *M* (*SD*) | *M* (*SD*) | *M* (*SD*) | Effect | *F* | *p* | η^2^_p_ | BF_10_ |  |
| IAT correct | .94 (.04) | .92 (.05) | .95 (.04) | .93 (.07) | D | 3.22 | .075 | .02 | 0.75 | Autistic = Nonautistic |
|  |  |  |  |  | G | 6.03 | .015 | .04 | 2.70 | Gender-referred > Cisgender |
|  |  |  |  |  | D × G | 0.01 | .931 | < .01 | 0.24 |  |
| IAT D | -0.13 (0.35) | 0.33 (0.30) | -0.18 (0.32) | 0.29 (0.27) | D | 0.85 | .358 | .01 | 0.23 | Autistic = Nonautistic |
|  |  |  |  |  | G | 76.93 | < .001 | .36 | > 30 | Gender-referred < Cisgender |
|  |  |  |  |  | D × G | 0.02 | .881 | < .01 | 0.23 |  |
| Explicit | 2.76 (0.52) | 2.83 (0.38) | 2.86 (0.36) | 2.89 (0.32) | D | 1.34 | .250 | .01 | 0.34 | Autistic = Nonautistic |
|  |  |  |  |  | G | 0.54 | .463 | < .01 | 0.23 | Gender-referred = Cisgender |
|  |  |  |  |  | D × G | 0.10 | .753 | < .01 | 0.23 |  |
| *Note.* *N* = 140. ANOVA = analysis of variance; D = child diagnostic status; G = child gender identity status; IAT correct = Implicit Association Test proportion of correct responses in the critical blocks; IAT D = Implicit Association Test D score. | | | | | | | | | | |

**Study 1: Results After Excluding Gender-Referred Participants Who Reported a Congruent Gender Identity**

Table S3 shows the mean scores on the IAT and the explicit task, along with the corresponding ANOVA results after excluding gender-referred participants whose experienced gender aligned with their assigned sex at birth (*n* = 6). As in the original unmatched sample, a 2 (diagnostic status: autistic/nonautistic) × 2 (gender identity status: gender-referred/cisgender) ANOVA on the proportion of correct responses in the critical IAT blocks yielded a significant main effect of gender identity status, indicating that gender-referred participants (*M* = .95; *SD* = .04) performed significantly better than cisgender participants (*M* = .93; *SD* = .06). Neither the main effect of diagnostic status nor the Diagnostic Status × Gender Identity Status interaction was significant.

Consistent with the original unmatched sample, a 2 (diagnostic status) × 2 (gender identity status) ANOVA on D scores yielded a significant main effect of gender identity status, with gender-referred participants (*M* = -0.19; *SD* = 0.32) scoring significantly lower than cisgender participants (*M* = 0.31; *SD* = 0.30). Neither the main effect of diagnostic status nor the interaction was significant. Furthermore, one-sample *t*-tests showed that, in keeping with the original unmatched sample, gender-referred participants scored significantly below zero, *t*(79) = -5.30, *p* < .001, *d* = -0.59, BF_10_ > 30, whereas cisgender participants scored significantly above zero, *t*(122) = 11.38, *p* < .001, *d* = 1.03, BF_10_ > 30.

Lastly, as in the original unmatched sample a 2 (diagnostic status: autistic/nonautistic) × 2 (gender identity status: gender-referred/cisgender) ANOVA on scores from the explicit measure yielded no significant main effects or interaction.

| **Table S3** | | | | | | | | | | |
| --- | --- | --- | --- | --- | --- | --- | --- | --- | --- | --- |
| *Mean Scores and ANOVA Results After Excluding Gendre-Referred Participants Who Reported a Congruent Gender Identity* | | | | | | | | | | |
| Variable | Autistic | | Nonautistic | | ANOVA | | | |  | Direction of Effects |
|  | Gender-referred | Cisgender | Gender-referred | Cisgender |  |  |  |  |  |  |
|  | *M* (*SD*) | *M* (*SD*) | *M* (*SD*) | *M* (*SD*) | Effect | *F* | *p* | η^2^_P_ | BF_10_ |  |
| IAT correct | .94 (.04) | .92 (.05) | .95 (.03) | .93 (.06) | D | 2.39 | .124 | .01 | 0.49 | Autistic = Nonautistic |
|  |  |  |  |  | G | 7.32 | .007 | .04 | 5.68 | Gender-referred > Cisgender |
|  |  |  |  |  | D × G | 0.05 | .823 | < .01 | 0.20 |  |
| IAT D | -0.14 (0.35) | 0.34 (0.29) | -0.22 (0.29) | 0.29 (0.31) | D | 2.00 | .159 | .01 | 0.33 | Autistic = Nonautistic |
|  |  |  |  |  | G | 122.80 | < .001 | .38 | > 30 | Gender-referred < Cisgender |
|  |  |  |  |  | D × G | 0.08 | .777 | < .01 | 0.22 |  |
| Explicit | 2.81 (0.43) | 2.84 (0.37) | 2.83 (0.38) | 2.81 (0.43) | D | 0.01 | .930 | < .01 | 0.16 | Autistic = Nonautistic |
|  |  |  |  |  | G | 0.01 | .930 | < .01 | 0.16 | Gender-referred = Cisgender |
|  |  |  |  |  | D × G | 0.15 | .703 | < .01 | 0.22 |  |
| *Note.* *N* = 203 (autistic gender-referred *n* = 34; autistic cisgender *n* = 55; nonautistic gender-referred *n* = 46; nonautistic cisgender *n* = 68). ANOVA = analysis of variance; D = child diagnostic status; G = child gender identity status; IAT correct = Implicit Association Test proportion of correct responses in the critical blocks; IAT D = Implicit Association Test D score. | | | | | | | | | | |

**Study 1: Results After Excluding Participants in the Process of an ASD Assessment**

Table S4 shows the mean scores on the IAT and the explicit task, along with the corresponding ANOVA results after excluding participants who were in the process of an ASD assessment (*n* = 5). As in the original unmatched sample, a 2 (diagnostic status: autistic/nonautistic) × 2 (gender identity status: gender-referred/cisgender) ANOVA on the proportion of correct responses in the critical IAT blocks yielded a significant main effect of gender identity status, indicating that gender-referred participants (*M* = .95; *SD* = .04) performed significantly better than cisgender participants (*M* = .93; *SD* = .06). Neither the main effect of diagnostic status nor the Diagnostic Status × Gender Identity Status interaction was significant.

Consistent with the original unmatched sample, a 2 (diagnostic status) × 2 (gender identity status) ANOVA on D scores yielded a significant main effect of gender identity status, with gender-referred participants (*M* = -0.17; *SD* = 0.33) scoring significantly lower than cisgender participants (*M* = 0.31; *SD* = 0.30). Neither the main effect of diagnostic status nor the interaction was significant. Furthermore, one-sample *t*-tests showed that, in keeping with the original unmatched sample, gender-referred participants scored significantly below zero, *t*(80) = -4.57, *p* < .001, *d* = -0.51, BF_10_ > 30, whereas cisgender participants scored significantly above zero, *t*(122) = 11.38, *p* < .001, *d* = 1.03, BF_10_ > 30.

Lastly, as in the original unmatched sample a 2 (diagnostic status: autistic/nonautistic) × 2 (gender identity status: gender-referred/cisgender) ANOVA on scores from the explicit measure yielded no significant main effects or interaction.

| **Table S4** | | | | | | | | | | |
| --- | --- | --- | --- | --- | --- | --- | --- | --- | --- | --- |
| *Mean Scores and ANOVA Results After Excluding Participants Undergoing ASD Assessment* | | | | | | | | | | |
| Variable | Autistic | | Nonautistic | | ANOVA | | | |  | Direction of Effects |
|  | Gender-referred | Cisgender | Gender-referred | Cisgender |  |  |  |  |  |  |
|  | *M* (*SD*) | *M* (*SD*) | *M* (*SD*) | *M* (*SD*) | Effect | *F* | *p* | η^2^_P_ | BF_10_ |  |
| IAT correct | .94 (.04) | .92 (.05) | .95 (.03) | .93 (.06) | D | 3.21 | .075 | .02 | 0.75 | Autistic = Nonautistic |
|  |  |  |  |  | G | 6.26 | .013 | .03 | 4.56 | Gender-referred > Cisgender |
|  |  |  |  |  | D × G | 0.21 | .644 | < .01 | 0.23 |  |
| IAT D | -0.12 (0.36) | 0.34 (0.29) | -0.20 (0.31) | 0.29 (0.31) | D | 1.77 | .185 | .01 | 0.44 | Autistic = Nonautistic |
|  |  |  |  |  | G | 107.62 | < .001 | .35 | > 30 | Gender-referred < Cisgender |
|  |  |  |  |  | D × G | 0.05 | .825 | < .01 | 0.23 |  |
| Explicit | 2.73 (0.54) | 2.84 (0.37) | 2.84 (0.37) | 2.81 (0.43) | D | 0.37 | .543 | < .01 | 0.16 | Autistic = Nonautistic |
|  |  |  |  |  | G | 0.36 | .547 | < .01 | 0.17 | Gender-referred = Cisgender |
|  |  |  |  |  | D × G | 1.12 | .292 | .01 | 0.68 |  |
| *Note.* *N* = 204 (autistic gender-referred *n* = 32; autistic cisgender *n* = 55; nonautistic gender-referred *n* = 49; nonautistic cisgender *n* = 68). ANOVA = analysis of variance; D = child diagnostic status; G = child gender identity status; IAT correct = Implicit Association Test proportion of correct responses in the critical blocks; IAT D = Implicit Association Test D score. | | | | | | | | | | |

**Study 1: Results After Matching Groups for IAT Performance**

To ensure that the between-group differences in the IAT D scores reported in the manuscript reflected differences in gender identity status (gender-referred/cisgender), rather than differences in the proportion of correct responses in the critical blocks, we matched groups on this variable using an R script (version 4.3.3; R Core Team, 2024). Groups were considered matched if *p*-values were greater than .05 and effect sizes were small.

To achieve this, we first excluded participants (*n* = 3) with error rates greater than 20% in the critical blocks of the IAT (Greenwald et al., 1998). Then, three phases of exclusion followed. First, we excluded participants with the highest performance in the gender-referred groups and those with the lowest performance in the cisgender groups. Next, we excluded participants with the highest performance in the nonautistic groups and those with the lowest performance in the autistic groups. Lastly, we excluded the participant with the highest performance in the nonautistic gender-referred group and the participant with the lowest performance in the autistic cisgender group. After this step, analyses showed that groups were matched for IAT performance. Matching statistics and ANOVA results are presented in Table S5.

As in the original unmatched sample, a 2 (diagnostic status) × 2 (gender identity status) ANOVA on D scores yielded a significant main effect of gender identity status, with gender-referred participants (*M* = -0.18; *SD* = 0.32) scoring significantly lower than cisgender participants (*M* = 0.32; *SD* = 0.30). Neither the main effect of diagnostic status nor the interaction was significant. Furthermore, as in the original unmatched sample, one-sample *t*-tests showed that gender-referred participants scored significantly below zero, *t*(80) = -4.79, *p* < .001, *d* = -0.53, BF_10_ > 30, whereas cisgender participants scored significantly above zero, *t*(114) = 11.44, *p* < .001, *d* = 1.07, BF_10_ > 30.

| **Table S5** | | | | | | | | | | |
| --- | --- | --- | --- | --- | --- | --- | --- | --- | --- | --- |
| *Mean Scores and ANOVA Results After Matching Groups for Percentage of Correct Responses in the IAT Critical Blocks* | | | | | | | | | | |
| Variable | Autistic | | Nonautistic | | ANOVA | | | |  | Direction of Effects |
|  | Gender-referred | Cisgender | Gender-referred | Cisgender |  |  |  |  |  |  |
|  | *M* (*SD*) | *M* (*SD*) | *M* (*SD*) | *M* (*SD*) | Effect | *F* | *p* | η^2^_P_ | BF_10_ |  |
| IAT correct | .94 (.04) | .93 (.04) | .95 (.03) | .94 (.04) | D | 3.03 | .083 | .02 | 0.66 | Autistic = Nonautistic |
|  |  |  |  |  | G | 2.94 | .088 | .02 | 0.67 | Gender-referred = Cisgender |
|  |  |  |  |  | D × G | 0.03 | .871 | < .01 | 0.22 |  |
|  |  |  |  |  |  |  |  |  |  |  |
| IAT D | -0.14 (0.35) | 0.35 (0.29) | -0.20 (0.31) | 0.29 (0.30) | D | 1.52 | .219 | .01 | 0.27 | Autistic = Nonautistic |
|  |  |  |  |  | G | 116.92 | < .001 | .38 | > 30 | Gender-referred < Cisgender |
|  |  |  |  |  | D × G | 0.00 | .964 | < .01 | 0.22 |  |
|  |  |  |  |  |  |  |  |  |  |  |
| *Note.* *N* = 196 (autistic gender-referred *n* = 35; autistic cisgender *n* = 51; nonautistic gender-referred *n* = 46; nonautistic cisgender *n* = 64). ANOVA = analysis of variance; D = child diagnostic status; G = child gender identity status; IAT correct = Implicit Association Test proportion of correct responses in the critical blocks; IAT D = Implicit Association Test D score. | | | | | | | | | | |

**Study 2: Results After Matching Groups for Assigned Sex at Birth and Age**

To ensure that the findings of Study 2 were not confounded by between-group differences in caregiver age and assigned sex at birth ratio, we matched groups on these variables using an R script (version 4.3.3; R Core Team, 2024). Groups were considered matched if *p*-values were greater than .05 and effect sizes were small. To achieve this, the youngest caregivers were gradually removed from each group, separately for participants assigned male at birth and participants assigned female at birth, until groups were balanced in *n* with the group of caregivers of autistic gender-referred youth. After this step, analysed showed that groups were matched for both age and assigned sex at birth (see Table S6).

As in the unmatched sample, one-sample *t*-tests showed that D scores differed significantly from zero in participants assigned female at birth (*n* = 190, *M* = 0.53, *SD* = 0.37), *t*(189) = 19.96, *p* < .001, *d* = 1.45, BF_10_ > 30. Although D scores among participants assigned male at birth (*n* = 4, *M* = -0.40, *SD* = 0.48) did not differ significantly from zero, *t*(3) = -1.66, *p* = .195, *d* = -0.83, BF_10_ = 0.95, the effect size remained large, as observed in the original sample. Furthermore, consistent with the original sample, an independent *t*-test showed that the difference in D score between sexes was significant, *t*(192) = 4.99, *p* < .001, *d* = 2.52, BF_10_ > 30, confirming that participants assigned female at birth demonstrated a stronger association with female gender groups, while participants assigned male at birth showed a stronger association with male gender groups. Also in keeping with the unmatched sample, a 2 (child diagnostic status: autistic/nonautistic) × 2 (child gender identity status: gender-referred/cisgender) ANOVA on IAT D scores yielded no significant main effects or interaction.

Just as in the unmatched sample, one-sample *t*-tests showed that self-reported strength of gender identity differed significantly from zero in participants assigned female at birth (*n* = 190, *M* = 4.50, *SD* = 1.44), *t*(189) = 43.19, *d* = 3.13, BF_10_ > 30, as well as participants assigned male at birth (*n* = 4, *M* = -3.79, *SD* = 0.98), t(3) = -7.70, *p* = .005, *d* = -3.85, BF_10_ = 11.86. An independent *t*-test showed that the difference between sexes was significant, *t*(192) = 11.47, *p* < .001, *d* = 5.80, BF_10_ > 30. Also in keeping with the unmatched sample, a 2 (child diagnostic status: autistic/nonautistic) × 2 (child gender identity status: gender-referred/cisgender) ANOVA on participants’ explicit task scores revealed a significant main effect of child gender identity status, with caregivers of the gender-referred youth (*M* = 4.27; *SD* = 1.63) reporting significantly lower gender-group identification than caregivers of cisgender youth (*M* = 4.71; *SD* = 1.17). Neither the main effect of child diagnostic status nor the interaction was significant.

| **Table S6** | | | | | | | | | | |
| --- | --- | --- | --- | --- | --- | --- | --- | --- | --- | --- |
| *Sample Characteristics and ANOVA Results for Age, IAT, and Explicit Task Performance* | | | | | | | | | | |
| Variable | Autistic | | Nonautistic | | ANOVA | | | |  | Direction of Effects |
|  | Gender-referred | Cisgender | Gender-referred | Cisgender |  |  |  |  |  |  |
|  | *n* = 49  (98% AFAB) | *n* = 49  (98% AFAB) | *n* = 47  (98% AFAB) | *n* = 49  (98% AFAB) |  |  |  |  |  |  |
|  | *M* (*SD*) | *M* (*SD*) | *M* (*SD*) | *M* (*SD*) | Effect | *F* | *p* | η^2^_p_ | BF_10_ |  |
| Age | 44.92 (4.62) | 43.31 (6.17) | 44.49 (6.91) | 43.69 (4.30) | D | 0.00 | .980 | < .01 | 0.16 | Autistic = Nonautistic |
|  |  |  |  |  | G | 2.25 | .135 | .01 | 0.45 | Gender-referred = Cisgender |
|  |  |  |  |  | D × G | 0.26 | .612 | < .01 | 0.23 |  |
| IAT correct | 0.97 (0.15) | 0.99 (0.14) | 0.97 (0.03) | 0.98 (0.02) | D | 0.41 | .524 | < .01 | 0.19 | Autistic = Nonautistic |
|  |  |  |  |  | G | 1.21 | .272 | .01 | 0.28 | Gender-referred = Cisgender |
|  |  |  |  |  | D × G | 0.20 | .652 | < .01 | 0.22 |  |
| IAT D | 0.45 (0.44) | 0.52 (0.39) | 0.53 (0.28) | 0.62 (0.33) | D | 3.10 | .080 | .02 | 0.68 | Autistic = Nonautistic |
|  |  |  |  |  | G | 2.13 | .146 | .01 | 0.43 | Gender-referred = Cisgender |
|  |  |  |  |  | D × G | 0.02 | .876 | < .01 | 0.22 |  |
| Explicit | 4.04 (1.98) | 4.74 (1.27) | 4.50 (1.14) | 4.67 (1.07) | D | 0.94 | .333 | .01 | 0.24 | Autistic = Nonautistic |
|  |  |  |  |  | G | 4.59 | .033 | .02 | 1.37 | Gender-referred < Cisgender |
|  |  |  |  |  | D × G | 1.66 | .199 | .01 | 0.43 |  |
| Note. *N* = 194. AFAB = assigned female at birth; ANOVA = analysis of variance; D = child diagnostic status; G = child gender identity status; IAT correct = Implicit Association Test proportion of correct responses in the critical blocks; IAT D = Implicit Association Test D score. | | | | | | | | | | |

| **Table S7**  *Study 1: Deviations for Pre-Registration* | | |
| --- | --- | --- |
| **Where?** | **What?** | **Why?** |
| Sample size | Final sample: 37 autistic gender-referred, 55 autistic cisgender, 49 nonautistic gender-referred, and 68 nonautistic cisgender, rather than 50 per group | - High attrition rate, the closure of the UK’s only national gender identity service, and general recruitment challenges - To maximise statistical power, additional cisgender participants recruited for matching purposes were included in the sample |
| Data collection | - Age range 7–16 years instead of 7–14 years - Use of social media to recruit both cisgender and gender-referred participants | To maximise the chances of achieving the target sample size, and consequently, ensure adequate statistical power to conduct the planned statistical analyses |
| Data exclusion | - Gender referred children undergoing an ASD assessment were included in the autistic gender clinic-referred group - Gender-referred children who reported an expressed gender that aligned with their assigned sex at birth were included in the gender-referred group | Due to difficulties meeting the target sample size for the gender-referred groups. |
| Data analysis | - Preliminary analyses using intergroup scoring were omitted for both the IAT and explicit gender identity task. | - Main analyses using ingroup scoring showed that cisgender participants identified with their assigned sex at birth, whereas gender-referred participants identified with the opposite binary sex. This confirms the IAT’s sensitivity to gender identity, rendering intergroup scoring redundant. - The pre-registration included an error: the explicit task does not permit calculation of intergroup scores. |

| **Table S8**  *Study 2: Deviations for Pre-Registration* | | |
| --- | --- | --- |
| **Where?** | **What?** | **Why?** |
| Sample size | Final sample: 49 autistic gender-referred, 54 autistic cisgender, 51 nonautistic gender-referred, and 63 nonautistic cisgender, rather than 50 per group | - High attrition rate, the closure of the UK’s only national gender identity service, and general recruitment challenges - To maximise statistical power, additional caregivers from the cisgender and nonautistic gender-referred groups, initially recruited for matching purposes, were included in the sample. |
| Data analysis | Between-group differences in sex ratio were examined conducting a series of Fisher’s exact tests, rather than a 3-way log-linear analysis. | - Due to the small number of assigned male at birth caregivers in the sample, some expected cell counts were below 5, so the planned analysis could not be conducted. |
